# A 11-year-old boy with Blastocystis hominis infection, presents as immune thrombocytopenia

**DOI:** 10.1186/s12959-024-00611-w

**Published:** 2024-04-29

**Authors:** Fajuan Tang, Dongqiong Xiao, Lin Chen, Xihong Li, Lina Qiao

**Affiliations:** 1 Department of Emergency, West China Second University Hospital, Sichuan University, Chengdu, 610041 Sichuan China; 2 Key Laboratory of Birth Defects and Related Diseases of Women and Children (Sichuan University), Ministry of Education, Chengdu, 610041 China; 3 Department of Pediatrics, West China Second University Hospital, Sichuan University, Chengdu, 610041 Sichuan China

**Keywords:** Immune thrombocytopenia, Blastocystis hominis, Cytokines

## Abstract

**Background:**

Some causes of first-line treatment failure for ITP are often closely related to infections. But parasitic infections are rarely mentioned and easily overlooked. The case is the first to describe a boy with immune thrombocytopenia associated with blastocystis hominis.

**Case presentation:**

The case involved a boy presenting with bleeding skin spots and ecchymosis and accompanied by intermittent epigastric pain and constipation. After a series of complete examinations, the platelet count was found to be decreased to 13 × 10^9^/L and immune thrombocytopenia was diagnosed. After first-line treatment with gamma globulin and prednisolone, the thrombocytopenia remained unchanged. Blastocystis hominis was subsequently found in the patient's stool and then the treatment of metronidazole was provided. One week later, the patient's thrombocytopenia was completely relieved. He was followed up for six months and was found to have recovered well.

**Conclusions:**

The screening for potential predisposing factors is very important for immune thrombocytopenia patients with poor response to first-line treatment, and the best treatment strategy should include the management of potential diseases.

## Introduction

Immune thrombocytopenia (ITP) is an acquired autoimmune disease characterized by low platelet counts (< 100 × 10^9^/L) and an increased risk of bleeding [1]. First-line therapy for ITP is are corticosteroids and intravenous immunoglobulins [2]. However, sometimes first-line treatment failure is observed in the patients. ITP can occur in isolation (primary ITP) or in association with a predisposing condition (secondary ITP) [1]. Some predisposing conditions are closely related to infections (such as viruses, bacteria, and Mycoplasma pneumoniae) [3, 4]. Parasitic infections are often rarely mentioned and therefore easily overlooked. Our case firstly describes a boy with ITP associated with Blastocystis hominis (BH) infection.

## Case presentation

**Fig. 1:**
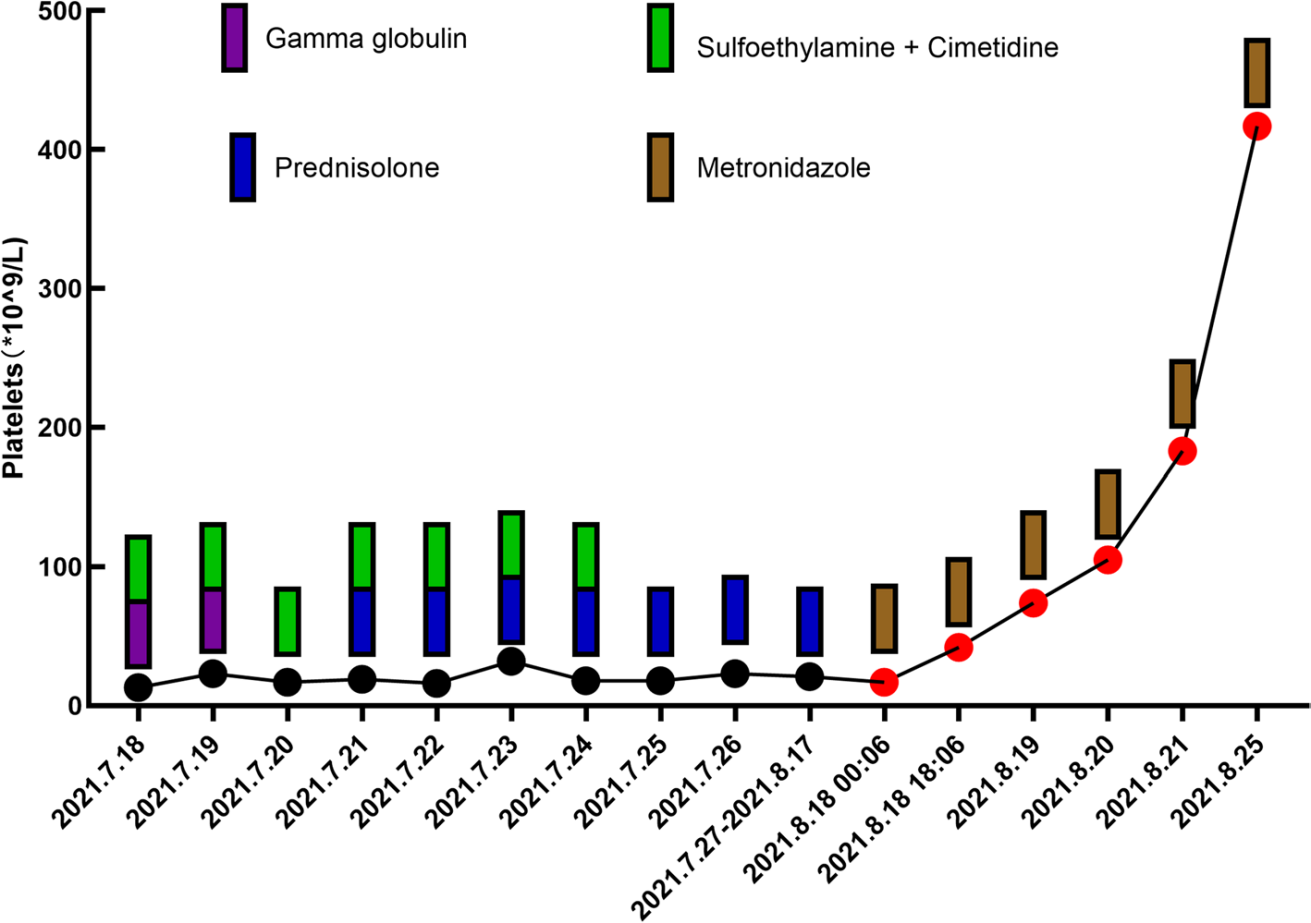
Platelet counts, presented by treatment, over time. The black dots represent platelet counts at local hospitals on different days, while the red dots represent platelet counts at our hospital. The rectangles above the dots represent the relevant medication on that day

The patient was an 11-year-old male who visited a local hospital in August 2021 after discovering skin bleeding spots and ecchymosis that had been present for 3 days. The boy had paroxysmal epigastric pain and constipation for two weeks before his presentation. He denied any recent history of respiratory infection or vaccination. The physical examination revealed hemorrhagic spots and ecchymosis on the skin of the calf. The superficial lymph nodes, liver, and spleen were not palpable or enlarged. Routine blood tests showed that the platelet count (PLT) was 13 × 10^9^/L; the white blood cell count, red blood cell count, and hemoglobin were normal (Table 1). ALT, AST, creatinine, urea, PT, APTT, and electrolytes were normal. B-scan ultrasound of the liver, gall bladder, pancreas, spleen, intestine, and appendix failed to identify any abnormalities. At the same time, the platelet IgG was tested, and the result was positive. The boy was considered to have ITP, and a series of treatments were instituted (Fig. 1). First, gamma globulin was administered at 1 g/kg. The next day, routine blood tests showed that the PLT count had increased to 23 × 10^9^/L. The gamma globulin infusion was administered again at the same dose. On the following day, the PLT count dropped to 17 × 10^9^/L. Considering this treatment failure, the local physician conducted screening for possible underlying infectious factors and immune diseases. On the following day, the PLT count was 19 × 10^9^/L, and a plain chest X-ray showed no abnormalities. Mycoplasma pneumoniae IgM, Chlamydia pneumoniae IgM, respiratory syncytial virus IgM, adenovirus IgM, and Coxsackie virus IgM antibodies were negative; HIV, HBV, HCV, HAV, and Helicobacter pylori antibodies were negative (Table 1). In addition, the boy's C13 urea breath test was negative and the urine test was normal. Autoantibody and thyroid function showed no abnormalities. In order to rule out acute leukemia and aplastic anemia, they tested the bone marrow examination. The bone marrow examination showed plate-producing megakaryocytopenia, which was consistent with thrombocytopenic bone marrow. According to these results, the patient was commenced on oral prednisolone at 1 mg/kg/d. Over the next twenty days, the PLT count was fluctuated around 21 × 10^9^/L. During the above treatment, the patient was also given an infusion of 10 mg/kg of sulfoethylamine (Because of a lot of skin bleeding spots) and 200 mg of cimetidine (Fig. 1). However, the boy continued reporting episodic abdominal pain and constipation, and the thrombocytopenia could not be relieved.

**Fig. 2:**
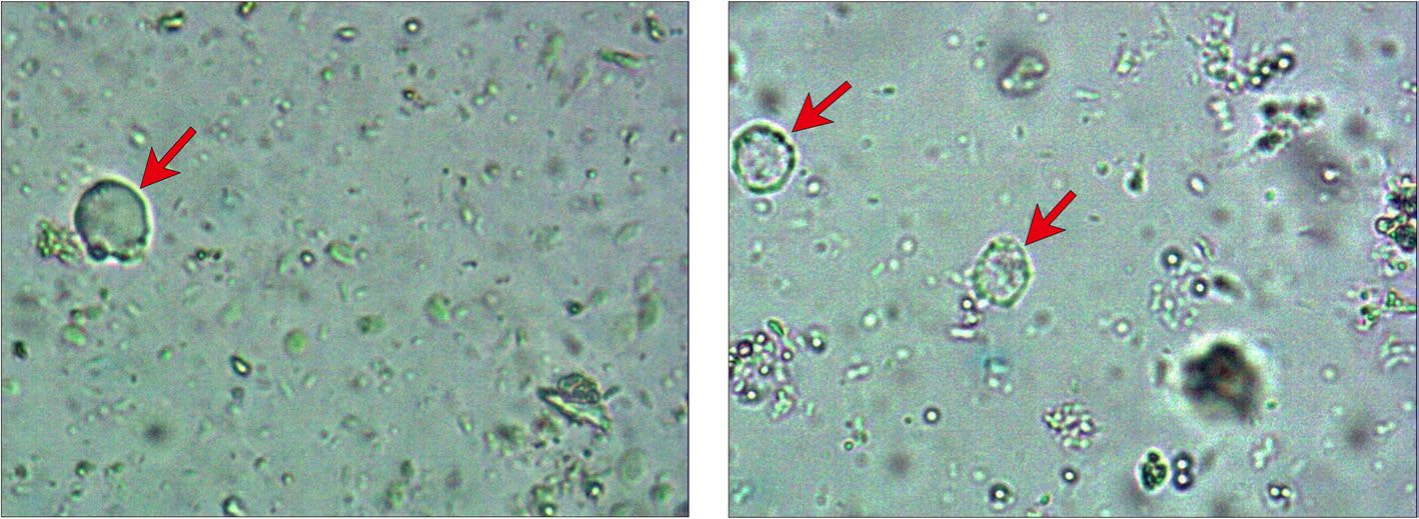
Blastocystis hominis in the stool as seen under a microscope. The vacuolar forms of blastocystis hominis were identified during the microscopic (× 200) examination of the boy's stool. The body is round, with a large clear vacuole in the center, there is a gap between the vacuole and the cell membrane, and several block nuclei (red arrows) are seen on the edge of the vacuole

At this point, the boy presented to our hospital for treatment. We found that the PLT count was 17 × 10^9^/L. Considering the failure of first-line treatment and the patient's accompanying gastrointestinal symptoms, we conducted a stool pathogen examination. BH was found in the stool (Fig. 2). Subsequently, we performed a DNA pathogenic microbial macro analysis of the boy's stool and blood. The results showed that BH infection was found in the stool and negative in the blood. In addition, we found that in the boy's blood the level of cytokines (IL-6, IL-17, IFN-γ) was increased (Table 1); TNF-α, IL-4, IL-8, IL-10, IL-5 and IL-2 were not significantly altered. Then, metronidazole was administered against BH. The following day, the PLT count increased to 42 × 10^9^/L and to 74 × 10^9^/L and 105 × 10^9^/L over the next two days. After 1 week of continuous oral metronidazole, the PLT count increased to 417 × 10^9^/L. At the same time, the abdominal pain and constipation were relieved. Re-examination of stool showed negative. The metronidazole was ceased and the patient’s PLT count was maintained at 350–420 × 10^9^/L during follow-up over the following six months.

**Table 1:** Routine lab test results

Lab tests	Results	Reference values
Routine blood		
White blood cell count	5.0	3.6–9.7 (× 10^9^/L)
Red blood cell count	4.87	3.82–5.5 (× 10^12^/L)
Hemoglobin	135	110–146 (g/L)
Platelet count	13	100–450 (× 10^9^/L)
Liver function		
Aspartate Transaminase	12	< 49 (U/L)
Alanine aminotransferase	34	< 40 (U/L)
Kidney function		
Ccreatinine	54	35.9–83.1 (umol/L)
Urea	4.31	3.2–8.2 (nmol/L)
Coagulation Function		
Prothrombin time	12.1	7.6–13.6 (second)
Activated partial thromboplastin time	25.9	18.1–38.1 (second)
Electrolytes		
Serum potassium	4.2	3.5–5.5 (mmol/L)
Serum sodium	137.6	132–146 (mmol/L)
Serum calcium	2.35	2.25–2.67 (mmol/L)
Serum magnesium	0.78	0.53–1.11 (mmol/L)
Thyroid function		
T3	1.53	1.29–3.0 (nmol/L)
T4	74.90	73.53–161.25 (nmol/L)
TSH	3.075	0.64–6.27 (mIU/L)
FT3	5.4	5.1–8.0 (pmol/L)
FT4	14.98	12.26–21.67 (pmol/L)
TGAb	61	< 60 (U/ml)
TPOAb	58	< 60 (U/ml)
Autoantibody	-	-
Pathogen		
Mycoplasma pneumonia IgM	-	-
Chlamydia pneumoniae IgM	-	-
Helicobacter pylori IgG	-	-
HIV antibody	-	-
Anti-HCV/HCV-cAg	-	-
HBsAg/HBeAg/HBV-PreS1	-	-
Anti-HBe/HBcAb-IgM	-	-
HAV-IgG/IgM	-	-
TP antibody	-	-
EB virus IgM/IgG	-	-
Respiratory Cytovirus IgM	-	-
Adenovirus IgM	-	-
Coxsackie virus IgM	-	-
C13 urea breath test	-	-
Cytokines		
IL-6	69.35	< 20.0 (pg/ml)
IL-17	83.21	< 20.6 (pg/ml)
IFN-γ	43.27	< 217.3 (pg/ml)
TNF-α	1.70	< 5.50 (pg/ml)
IL-4	4.19	< 12.90 (pg/ml)
IL-8	12.63	< 21.40 (pg/ml)
IL-10	3.86	< 5.90 (pg/ml)
IL-5	< 1.50	< 3.40 (pg/ml)
IL-2	4.95	< 11.40 (pg/ml)

“-” indicates a negative result

## Discussion

BH, an intestinal parasitic protozoan, exhibits primarily vacuolar, granular, and amoebic features [5]. Clinical presentations of BH infection include abdominal pain and diarrhea. A minority of patients experience symptoms such as anorexia, abdominal distension, constipation, and stomach pain [6]. BH was historically considered to be clinically inconsequential, requiring little attention. However, has emerged as an opportunistic pathogen with relevance to intestinal disease. For instance, a systematic review suggested that intestinal colonization by BH may exacerbate colorectal cancer by altering the host immune response and increasing oxidative damage [7]. Additionally, BH is implicated in the development of irritable bowel syndrome through its influence on serum IgA levels [8]. Moreover, BH infection has been linked with several extraintestinal diseases [9, 10]. In a case involving a boy with Henoch-Schönlein Purpura, who experienced recurrent abdominal pain and rash after steroid treatment, BH was detected in the stool; symptoms improved following BH eradication with a compound triazole [9]. Additionally, BH contributes to the occurrence of Hashimoto's thyroiditis by engaging in immune responses, and BH eradication has shown promise in ameliorating Hashimoto's thyroiditis [10, 11]. Notably, in our case, we detected BH in the patient's stool.

In our case, the boy's platelet count did not improve following first-line treatment. Intriguingly, only after metronidazole was administered to eradicate BH did we observe a recovery in his platelet count. These findings suggest an unidentified relationship between BH infection and the pathogenesis of ITP. Although the mechanism of BH infection in some immune diseases has been elucidated, this is the first instance that BH infection was identified in the stool of patients with ITP. Consequently, the precise manner in which BH contributes to the onset of ITP remains unclear.

The disruption of intestinal microflora is a critical mechanism in understanding ITP. A study involving 16 children with ITP showed an increased abundance of Bacteroides and actinomyces in their stool compared to healthy children. This shift in population potentially contributes to the development of ITP by regulating IgG levels [12]. Remarkably, a study comparing gut flora in BH positive and BH-negative individuals reported alterations in the abundance of Bacteroides and Firmicutes in the former, resembling patterns seen in dysbiotic flora [13]. In addition, another study demonstrated that the presence of BH was associated with increased gut bacterial diversity, with a negative correlation observed with bacteroides levels [14]. This leads us to speculate that BH may play a role in the onset of ITP by influencing the equilibrium of intestinal microflora and the abundance of specific intestinal bacteria. However, since our patient did not undergo a complete stool microbiota examination, further research is necessary to test this relationship.

ITP patients exhibit an imbalance of CD4 + T cell subsets and abnormal secretion of associated cytokines [15]. CD4 + T cells differentiate into TH1 cells (IFN-γ, IL-2, TNF-β) and TH2 cells (IL-4, IL-5, IL-6, IL-10, IL-13), while TNF-α is secreted by macrophages. Collectively they play an important role in the pathogenesis of ITP. Additionally, Th17 cells (IL-17A, IL-17F, IL-21, and IL-22) are known to play an inflammatory role in mediating autoimmune diseases.[15].

One study showed elevated levels of IL-6, IL-17, and IFN-γ in the serum of ITP patients, along with decreased levels of IL-4 and TGF-β [16]. In our patient, we observed an increase in IL-6, IL-17, and IFN-γ, while TNF-α, IL-4, IL-8, IL-10, IL-5 and IL-2 were not significantly altered. It has been shown that BH can modulate immune responses, leading to the activation of immune cells and an increase in various interleukins [17]. The diverse BH subtypes may exert varying effects on intestinal flora and cytokines, resulting in distinct outcomes. One study suggested that the BH3 subtype shows greater therapeutic potential than others, promoting an increase in IFN-γ levels and inducing inflammatory responses [18]. IL-17, a key cytokine involved in regulating autoimmune disease, is secreted by Th17 cells and CD8-positive T cells, both of which are increased in ITP [19]. In cases of steroid-refractory ITP, there is an elevated IL-17 expression [20]. Studies have demonstrated high IL-17 expression in the intestinal mucosa of BH-infected mice [21]. The BH7 subtype has been shown to trigger ERK and JNK pathways in vitro, thereby influencing the expression of pro-inflammatory cytokines in macrophages, including IL-1β, IL-6, and TNF-α [22]*.* Based on these findings, we speculate that the observed cytokine abnormalities may be a potential mechanism by which BH infection could contribute to the development of ITP. Unfortunately, we did not go further to conduct a genetic classification. In addition, because the father did not agree to draw the patient's blood again, no results were obtained on cytokine levels after ITP remission.

In short, the exact mechanism of thrombocytopenia due to BH infection in this patient is unclear. But this interesting case reminds us of the importance of screening for potential predisposing factors in all ITP patients especially in those who did not respond well to the standard first-line treatment. The optimal treatment strategy for secondary ITP should include the management of the underlying disease.

## Data Availability

No datasets were generated or analysed during the current study.

## Abbreviations

ITP: Immune thrombocytopenia
BH: Blastocystis hominis
PLT: Platelet count
